# To Our Readers

**Published:** 1887-12

**Authors:** 


### TO OUR READERS.

The present number of our journal closes the volume for 1887. We are glad to say, despite the constantly increasing competition resulting from the large number of new and cheap journals that have been started in the last few years, that the SOUTHERN MEDICAL RECORD has held its own, and is prospering. As a practical journal of medium size, and moderate cost, it has proven itself well adapted to the wants of the busy practitioner, and numerous names on our list have taken the RECORD from its beginning in 1870, and adhere to it as the favorite journal among all that are published in the country North or South.

It has been the constant aim of the editors to make the RECORD instructive, useful and practical in all departmenis, and it is their intention to make it even more so in the future. That we may be the better able to do this, we earnestly request our readers to settle all past dues promptly, and renew their subscriptions for the next year.

And we here remind you of our liberal offer to give club rates, on renewal, to every one who will send us a new subscriber. That is, we will send two copies of the RECORD for $3.00, or, $1 50 each for yourself and for any new name you may send us. We ask every one of our subscribers to avail themselves of this offer, and in thus aiding us to extend our list you will do yourself a benefit by helping us in the work of improving your journal, and will also confer upon us a special favor as friends and brethren in the profession.

